# The Health of the Dentist—Means of Preserving

**Published:** 1869-04

**Authors:** H. M’Cullum


### THE HEALTH OF THE DENTIST—MEANS OF PRESERVING.

BY H. M'CULLUM, D. D. S.

(Continued from page 120.)

If the writer of this essay understands himself and his subject, the object of this paper is to so advise the Dental practitioner as to enable him to avoid the influences that would be likely to affect his health injuriously, while engaged in the duties of his chosen profession.

In a word, the object of this essay is to aid such as regard their health as a matter of primary importance, in prosecuting the duties of an honorable and beneficent profession in such a manner as to avoid the dangers to which they are liable, while engaged in that vocation.

If this end can be achieved, it is certainly a most worthy and desirable one ; where it can not, and where the D. D. S. has not sufficient faith in the "*vis medicatrix naturæ*" to warrant him in seeking aid in that direction, it is better that he call to his aid some medical friend, in whose professional skill he has the necessary confidence. I am one of that class who would not presume to write a prescription for an individual, of whose symptoms of disease and peculiarities of constitution I had every thing to learn.

Ignorance is but a blind guide in those who offer advice, as well as those who seek it.

If one has not a sufficient understanding of the laws of hygeine to enable him to avoid disease while in health, he is poorly prepared to pilot himself back to health, after disease has overtaken him ; and the most favorable time to gain the required knowledge, is not while suffering the pain and distress incident to disease, but rather while enjoying the vigor and sprightliness of health.

The greatest experts in medical science call some of their brother physicians to their aid if they become sick, and this is no doubt the best they can do under the circumstances, to seek the aid of the friends, in whose intelligent sympathy they have the highest confidence, when sick. But seek knowledge during health. There is one more condition to health that we may not pass unnoticed, viz.: the necessary alternations between activity and rest.

This natural law of the animal organization is as constantly prevalent, as imperative and as all pervading in its demands, as the necessity of food or respiration; this is not merely a general law, but applies to each member of the organism, separately and distinctly. Activity is no more essential to health than a due interval of repose, not only the voluntary but the involuntary actions or organs, require their appointed seasons of increased and diminished activity. Vegetable life is also characterized by alternate periods or seasons, of active development and wintry suspension of vital processes.

For the Dentist, and all others who are engaged in what are termed sedentary employments, it is important that they attend to the exercising of those members or faculties that are not brought into exercise (if there be any such), during the performance of their regular professional labors, that they take constant care to allow a sufficient time regularly to sleep, and recover from that feeling of lassitude and prostration that follows severe or protracted effort, and that at the same time they guard against the opposite extreme, viz.: a tendency to permit the necessity for rest lead to habits of idleness, listlessness, or downright laziness.

The importance of a sufficient amount of time, set apart at regular diurnal intervals, for sound and uninterrupted sleep, can not well be over-estimated; the most favorable conditions to sleep are, a dark room, with free ventilation, a mild temperature, firm bed and light covering, the stomach unincumbered with undigested food, a mind free from anxious care, a conscience at peace and void of offense, the pulse free and regular, but subsiding after the toils and exciting activities of the day.

Thus far we have considered the Dentist merely as an individual member of the human family, we can only touch on his social and moral relations, though the external relations may exert an important influence on the health, the subject is too vast, with complications too intricate and too widely diffused, to permit its being treated fully in a treatise like the present.

But there is one point in man's external relations, that we must not pass unnoticed. There is a claim on his careful and constant attention that he may not ignore or neglect with impunity. There is a power higher than his own, that guides his destiny, if it be guided safely and successfully. No one, however wise, or strong, or cunning, maintains an independent existence; but whatever of wisdom, or strength, or skill he may lay claim to, has been received from the great Creator, the rightful sovereign of all things, from whom every good and every perfect gift is derived; and he can only retain, or use such advantages or gifts as may be placed within his reach, in conformity with certain fixed and specific laws, that must ever limit and control all human actions; and an understanding of these laws comprehends the whole sum of human knowledge. A conformity to them secures the highest attainable degree of human success, of health and happiness. The first lesson, then, in a manual of health is, of necessity, it must be an acquaintance with the laws that the Creator has enacted and established for the government of man's physical nature.

Now, don't be alarmed my worthy friends. I do not propose to set myself up as the champion of any phase of sectarian bigotry, or ecclesiastical dogmatism, but hope to be indulged in a few remarks on the propriety of studying the divine will as such, as it is published in one volume, viz: the great volume of nature;* that we accustom ourselves to regard a knowledge of these laws as an indispensable condition to success in every enterprise in life, and especially when we seek the preservation or restoration of our health. All our actions as men are under the control of the will, guided by the understanding. If the understanding be at fault, the whole performance proves a failure. As rational beings our actions must be directed to the attainment of some desirable end, to the accomplishment of some worthy purpose. The divine law limits and fixes the relation or connection between causes and effects, or actions and their consequences in all things. No one can accomplish any worthy object, only in the way of obedience to the divine will. If we would co-operate with another, we must first understand the design of the intellect that projected the operation. We can only follow or accompany another, so far as we understand them. To act in harmony with the divine will it is necessary that we understand what that will is. This is as plain and explicit a statement of the case as we know how to make.

As social beings we are bound to seek and strive to promote the best interests of the community in which we live. Our own individual welfare is inseparably connected with the elevation, the purity and rectitude of the individuals composing the society of which we form a part. As moral, accountable beings we can not appropriately express the gratitude, reverence and devotion due to the author of our existence, otherwise than by an earnest effort to understand the purposes of his will respecting us, and an honest, persistent endeavor to perform the task assigned us in unwavering faith and unswerving integrity.

Some may suppose that it requires the eye of a fanatic to

*Not to the exclusion of Revelation]

see any connection between physical health and moral rectitude, or social propriety. We can not quite consent to be held responsible for all the errors that may be met with in public opinion, or the gaps that may be found in general intelligence. If all men were fully and correctly informed, a great number of monstrous and some very minute errors would be corrected, and the necessity for such a tract as this would be done away.

There are thousands to whose understanding the motion of the hands and feet would appear as a self evident fact, to whose individual perceptions the circulation of the blood would never be revealed, and still the proofs of the latter fact, to those who examine them intelligently, are quite as clear and conclusive as the former. There are, no doubt, truths that to one mind appear so plain that any argument offered in their support would appear as a superfluity, that his next neighbor could only regard as a fable or a falsehood. Must the incredulity or stupidity of the latter overthrow the faith of the former?

Though all men may have been created equal, politically considered, still there is almost an infinite diversity of characteristics and capabilities, when regarded as individuals, physically, socially, mentally or morally. As men approximate toward the character and condition of the lower animals, their physical status is less affected by moral and social influences, and as the intellectual and moral predominates, they are more liable to suffer in physical health from causes that assail them through the higher departments of their nature. As the moral, social and mental elements form a part of every true human character, and, as we are writing expressly for the benefit of humanity, we trust a few words of advice as to the care and management of the mental, moral and social relations as pertinent to the subject.

Men that desire good health should aim to secure peace of mind, a quiet conscience, unfaltering faith in the protecting care of omnipotent power and unerring wisdom, a ready acquiescence in the awards and behests of divine goodness; in a word, freedom from care, fear or regret, from whatever source or of whatever kind. In the next place they should take care that their domestic relations and interests are duly arranged and regulated. Whether man be universally admitted as a religious animal, no one can doubt the existence of the social, the domestic element in his nature, or lightly estimate its importance. Probably there is no external influence that will, in a large majority of cases, exert a more highly salutary and sanitary influence than the keeping the domestic relations under proper directions, regulations and limitations. No adverse external circumstance more certainly or speedily undermines and overthrows the health and peace than deranged, misplaced or misdirected domestic relations and affections. Some individuals are so constituted as to forego the domestic relations with impunity, but by far the greater number speedily fall into some fatal disease, when they see all hope and prospect of domestic and social happiness cut off. We here repeat the assertion, that all the faculties demand their full and legitimate exercise, under the impulse of their legitimate and appropriate stimulus, and every deviation from this requirement involves, first, functional and ultimately organic derangement, and while the moral sentiments, and social and domestic affections form composite elements in the human character, we can never permit them to run wild or hibernate too long with impunity. And as individuals approximate more and more nearly to the correct standard of humanity, the operation of those external influences become more deeply and extensively prevalent. True, there may be some whose susceptibility to external influences is in excess, but in a large majority of cases the merely animal selfish characteristics greatly preponderate.

I hope to be pardoned for having dwelt on this point somewhat at length, as I have labored under the impression that its importance is too often disregarded.

Permit me now to recapitulate briefly. The first matter that claims the attention of the honest, earnest seeker after health is, that he gain a full and correct understanding of the conditions on which health is obtained and preserved, of the laws that govern the physical nature of mankind, and, also, his own individual relations to health and tendency toward disease. It is important that he secure a regular supply of sound, healthy food, selected and prepared with due regard to his external surroundings; aye, occupation and constitutional peculiarities, &c., and when the claims of social etiquette or pecuniary interest conflict with his regular periods of eating, let the etiquette and the interest, with any other illmannered juveniles *wait*, while he takes his "daily bread" regularly, quietly, cheerfully and temperately.

It is also important that he avoid the necessity of breathing an impure or vitiated atmosphere; that in the construction and appointments of his habitation and place of business, and in the necessary contact and association with his fellow men, he avoid, as far as practicable, all disagreeable odors, all obnoxious vapors; that in his individual habits he accustom himself to a liberal share of outdoor exercise, without too much regard to the weather, without too careful avoidance of a little rain or sunshine, but careful to shun unclean localities, and absolutely to forego all kinds of filthy practices. Although the circulation is not under the direct control of the will, it is readily affected by actions and influences that are; as, likewise, by the emotions of hope, fear, joy, grief, mirth or despondency, which are not at all times under the direct control of the will.

The circulation, considered with regard to the force, frequency and regularity of its pulsations, may be taken as the most reliable index to the present vital conditions. But the most important practical idea in regard to the circulation, connects itself with the fact that it is controlled in its force and direction by the voluntary actions, that being the medium or channel through which the elements of nutrition are distributed to the various parts of the organism. We understand how men are developed and strengthened in whatever direction the voluntary actions indicate.

With regard to the nervous system, which unites all other parts in one individuality, and also connects us with the outside universe. Its multifarious capabilities are quickened, extended and strengthened in whatever direction the voluntary actions demand or direct the exercise of its functions Thus, all the different members or faculties are strengthened and endowed with greater activity, as the will, through the mysterious agency of the nerve force, directs the circulation.

To those who estimate health at its due value, it should be a leading consideration that the faculties be all trained to observe their due limits; that all the organs be duly and regularly exercised on their respectively appropriate objects, and that thus the vital operations or functions be regularly conducted or exercised in a healthy channel. Health and disease, like all other vital activities, may be the result of habit; or, in other words, may for a time continue in active operation after the apparent cause that first called them into activity has subsided.

A few words of special advice to Dentists and all others who desire to preserve or improve their health, and we close.

After you have learned to know yourself correctly, place a special guard over every weak point. If your digestion be slow or imperfect, use your best discrimination in the selection and preparation of your food; let no social influence or individual appetite betray you into any indiscretion in this direction. If your lungs be weak or irritable, never venture into an impure atmosphere. A large company in a close room should always be avoided. The smoke from coal and tobacco are a source of injury to thousands; the result of combustion every-where, whatever changes the constituent elements of the atmosphere from the normal proportions, tends to render the atmosphere unfit for the purposes of respiration, and especially dangerous to weak or diseased lungs.

Indeed, you can not oppress or abuse the stronger faculties with safety. Suppose, for instance, the digestive system be overtasked; if the lungs be healthy and vigorous, they will come to the rescue. A part of the superabundant carbon is consumed in the lungs, furnishing an increased temperature that favors perspiration and increased activity of the circulation, all combining to dispose of the undue amount of food with the least possible injury; whereas, if the lungs be weak the extra duty imposed by the stomach prostrates their feeble strength, obstructs and embarrasses their action, and straightway they fall into active disease; and this rule holds good throughout, that if we abuse or misuse one of the members, all the members suffer with it. There is one other admonition that must not be overlooked, viz: that those who desire safe and enduring health, must not rely on nervous stimulants, anodynes narcotics, &c. The demand for stimulants arises from an embarrassed or dissatisfied condition of the vital consciousness, growing awry the result of exercising some of the faculties inordinately, while others are left in idleness, to grow dwarfish and misshapen, for want of proper exercise and consequent development. There is a consciousness of deficiency or defectiveness, and the nervous system grasps at whatever arouses it to activity, as a drowning man clutches a straw.

Almost all heathens, whether savage or civilized, manifest an intuitive proclivity to seek enjoyment or excitement in the use of narcotic poisons, and none other but heathens in this or any other age, country or community. No candid votary of sound health will long be misled in this direction.

A competent military chief will not long wage war on his own sentinels, because they may sometimes disturb his repose. He well knows that his present safety and future success is too largely dependent on their vigilance and fidelity. The sense of discomfort that clamors for the gratification of a depraved or vicious appetite, is the alarm raised by the sentinels that the commander-in-chief of the life forces has appointed and stationed to guard the approaches to life's citadel, and the employment of narcotic poisons can not repel the adversary, but only silence the sentinels.

No one who estimates his life and health at their true value can afford to blunt his physical sensibilities with the paralizing influences of alcohol, tobacco or opium. Coffee and tea are also inimical to health and life, and the policy that includes them in our daily bill of fare is kindred to that which would incorporate traitors and rebels in a company of life guards.

A sense of pain, a consciousness of discomfort is the first indication of returning health, if health ever will or may return, and when the nervous consciousness is destroyed, all hope of a return to health is lost. Consciousness is one of the conditions of animal life, and full, correct, clear consciousness is a condition of good health. No one can justly claim to enjoy good health while they rely on the aid of some artificial stimulant to urge or spur the vital organs to the performance of their legitimate functions, and no one is warranted in regarding his health as in a safe condition while he suffers himself to tamper with these agents.

It was not the design, in the preparation of this paper, to say every thing that might be said on the subject of health, but rather to discuss some of the most prominent points, to refer to some of the fundamental principles of hygiene and pathology, and occasionally point to some of the landmarks for the better guidance and assurance of such as may look to these pages for assistance in regaining or preserving their health. Remember, Dentists are but men in all that relates to life or health.

